# Experiments push the limits of micromagnetic SANS theory

**DOI:** 10.1107/S2052252523005663

**Published:** 2023-06-28

**Authors:** Dieter Lott

**Affiliations:** a Helmholtz-Zentrum Hereon, Max-Planck-Strasse 1, Geesthacht, 21502, Germany

**Keywords:** small-angle neutron scattering, magnetism, high-pressure torsion, nano­crystalline materials, materials science, magnetic scattering

## Abstract

Commentary is provided on recent magnetic SANS experiments on highly inhomogeneous high-pressure-torsion manufactured metals. The ensuing progress in the theoretical description of magnetic SANS using micromagnetic theory is highlighted.

Progress in nearly all scientific areas is dominated by two main ingredients, the development of experimental methods that allow us to observe and investigate phenomena in the desired quality and precision, and the theoretical framework that delivers the ideas of the physical background giving a reasonable explanation of the experimentally observed quantities. Without the latter, it is nearly impossible to advance our understanding of the incredibly rich phenomena that we face in science and that help us to create new methods and ideas to feed technological, social and philosophical progress. However, the two main contributors to scientific progress are in general not isolated from each other, as it often may lead to a dead end if one observes phenomena where there is no potential explanation for a broader understanding, or a theory that has no counterpart in reality. Ideally, experiment and theory work in tandem as dynamic driving forces for each other. There may be an idea that triggers an experimental investigation to check its validity, or the observation of an unknown phenomenon that forces one to extend or even modify the current theory in a particular field. Looking at numerous examples in the past and the present, the ideal situation is realized when both parts are able to inspire their counterpart leading to successive improvements.

The article ‘Effect of annealing on the magnetic microstructure of high-pressure torsion iron: the relevance of high-order contributions to the magnetic small-angle neutron scattering cross section’ by Ma­thias Bersweiler *et al.* (2023 ▸), which is published in this issue of **IUCrJ** , represents a successful iteration of this process in the field of magnetic SANS. It is dedicated to the study of the magnetic properties of high-pressure-torsion (HPT) manufactured Fe using a wide spectrum of well developed experimental methods: magnetometry, X-ray diffraction, electron backscattering diffraction and, of course, small-angle neutron scattering (SANS). Recent advances in micromagnetic SANS theory (Metlov & Michels, 2015 ▸) extended to textured ferromagnets (Zaporozhets *et al.*, 2022 ▸) have opened up a way of extracting the global magnetic texture parameters from the magnetic SANS data in concert with the information obtained from the other employed magnetic analysis techniques. This recent theoretical development lifts an important limitation, which is the key to a better understanding of the highly relevant material class of HPT metals and the influence of the post-annealing process.

SANS is a unique experimental method that enables the exploration of the microstructure of a wide range of material classes that are relevant for industrial use and applied extensively in functional materials. In contrast to various forms of microscopy, neutrons penetrate deeply into matter, which permits the destructive-free investigation of the bulk properties of specimens. Since the neutrons possess a magnetic spin, their scattering is also sensitive to the magnetic properties, opening up a way to characterize the complex spin arrangements in the bulk of the samples. It can be thought of as a form of magnetometry, which characterizes bulk average magnetic properties, but with the sensitivity to the fine details (in the form of averaged amplitudes of spatial Fourier expansion coefficients) of the magnetization distribution on the mesoscale. Since the phase of the Fourier components is lost and only their bulk-averaged combination (not each of them separately) is measured, it is difficult (if not impossible) to directly reconstruct the magnetization distribution itself. Yet, many integral characteristics of the samples can be accessed in the form of various correlation functions, which can be connected to the details of the material treatment and/or stage of degradation. This connection is done through theoretical models that can be applied to the simulation and fitting of SANS data, *e.g.* two-dimensional detector images or one-dimensional cuts along specific directions in **q**-space. In particular, for investigations of the magnetic microstructure in the field of functional and applied materials, the interpretation opportunities of the SANS data have been drastically advanced in the last decade by several groups, in particular by the group of Andreas Michels (Department of Physics and Materials Science, University of Luxembourg). Initially starting from a simplistic model assuming homogeneously magnetized domains by using the classical particle-matrix concept, the modeling of the data was transformed into an approach using the continuum theory of micromagnetics. This methodology allows one to include spin-misalignment arrangements as well as additional terms that may have a significant impact on the magnetic microstructure, *e.g.* the Dzyaloshinshkii–Moriya interaction that plays an important role in various magnetic nanostructures lacking space inversion symmetry. It has proven to be an extremely powerful theory for gaining fundamental insights into the magnetic configuration of the investigated materials.

In the article by Bersweiler *et al.* (2023 ▸) the unpolarized SANS technique is used to obtain profound details on the magnetic microstructure of HPT-manufactured Fe undergoing a post-annealing process at different temperatures. The HPT method combined with post-processing techniques (such as annealing), while preserving the chemical composition of the sample, substantially changes the structure on micro- and nanoscales. These changes can range from a simple refining of the grains, up to a complete amorphization of the material. SANS is the perfect tool for studying samples of such a complex morphology. The paper illustrates experimentally, supported by the existing theory, how one is able to separate the magnetic from the nuclear contributions and obtain a detailed picture of the magnetic configuration, complemented by the results of magnetometry, X-ray diffraction and electron backscattering. An understanding of the magnetic grain distribution, in particular, is essential to study the effects of HPT and its post-processing. Fig. 1 ▸(*a*) shows the strong impact of the post-annealing effect on the grain size of HPT Fe. By the detailed evaluation of the purely magnetic SANS cross section, a global anisotropy factor can be determined that is slightly larger than the (expected) magnetocrystalline anisotropy value for bulk Fe, supporting the existence of an induced magnetoelastic anisotropy due to the HPT straining process. The careful fitting process of the magnetic SANS data revealed significant deviations that unambiguously demonstrate the presence of nonnegligible higher-order scattering contributions for the HPT samples. These deviations can be attributed to the high inhomogeneity of the ferromagnetic HPT samples and require an extension of the current micromagnetic SANS theory considering higher-order terms.

The work demonstrates how a sophisticated and well developed theory not only gives unique insights into the magnetic microstructure but also allows, by testing its limits on carefully recorded and reduced experimental data, for a steady development of the theory to untangle details of the fascinating magnetic properties, here in particular of the HPT process of metals. The goal will be to gain an even more profound understanding of the impact of the manufacturing processes on the magnetic microstructure by including even higher-order terms in the underlying magnetic SANS theory. This will not only significantly improve the understanding of the microstructural picture in these processes, but it may even allow the tailoring of the process itself, if the mechanism (pressure and post-annealing treatment) leading to the highly inhomogeneous microstructure is completely understood.

## Figures and Tables

**Figure 1:**
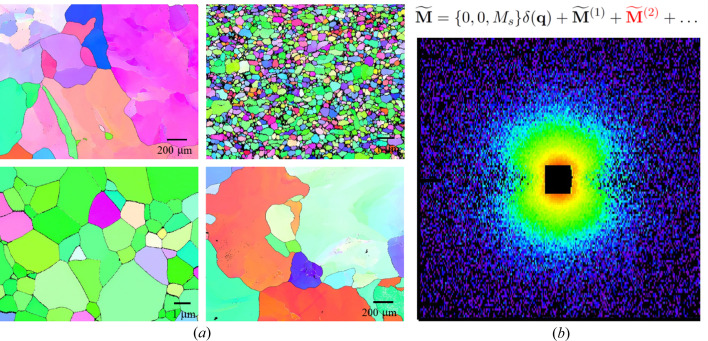
(*a*) Inverse pole figure maps of HPT Fe and non-deformed Fe obtained using EBSD are shown (Bersweiler *et al.*, 2023 ▸). Uniformly colored areas represent regions of the same (or nearly the same) crystallographic orientation. The influence of the post-annealing effect is clearly demonstrated, resulting in the different grain sizes. (*b*) Purely magnetic SANS cross section of HPT Ni at 1 T.
